# How to Live with the Enemy: Understanding Tolerance to Parasites

**DOI:** 10.1371/journal.pbio.1001989

**Published:** 2014-11-04

**Authors:** Lars Råberg

**Affiliations:** Functional Zoology, Department of Biology, Lund University, Lund, Sweden

## Abstract

How do we defend ourselves against pathogenic microbes and other parasites infecting us? Research on defence against parasites has traditionally focused on resistance—the ability to prevent infection or limit parasite replication. The genetics, physiology, and evolutionary ecology of such traits are now relatively well understood. During the last few years it has been realized that another, conceptually different type of defence also plays an important role in animal host–parasite interactions. This type of defence is called tolerance, and can be defined as the ability to limit the health effects of parasites without preventing infection or controlling parasite replication. Our understanding of the causes and consequences of variation in tolerance is, however, still rudimentary. Three recent studies shed light on these questions. In a study of HIV in humans, Regoes et al. show that an MHC class I gene affects not only resistance (as previously known) but also tolerance. In a study of voles, Jackson et al. identify a transcription factor mediating age differences in tolerance to macroparasites. Finally, Hayward et al. demonstrate that tolerance to intestinal parasites in sheep is under positive directional selection, but that most of the variation is environmentally induced rather than heritable. These studies increase our knowledge of the genetic and physiological sources of variation in tolerance, and how this variation affects Darwinian fitness. In addition, they illustrate different approaches to untangle tolerance from other factors determining the health effects of infectious disease.

## Abstract 2

This Primer examines the phenomenon of infection tolerance and discusses three recent *PLOS Biology* research articles about the causes and consequences of variation in tolerance in natural populations.

Most organisms are hosts to a wide diversity of pathogenic microbes and other parasites, which can have severe effects on host survival or other aspects of evolutionary fitness. For example, there are 1,407 different types of parasites infecting humans [1], and some of these cause infectious diseases that are leading causes of mortality (e.g., AIDS, malaria, and tuberculosis) [2]. The situation is similar for most other organisms, from prokaryotes to vertebrates. Infectious disease is therefore undoubtedly an important ecological and evolutionary force in all branches of the tree of life.

Natural selection by parasites has resulted in the evolution of a range of host defence mechanisms. The most well-known are those preventing infection or controlling parasite growth, that is, mechanisms that enhance host resistance. In animals, resistance mechanisms include for example epithelial mucus, anti-microbial peptides, phagocytic cells, and cytotoxic T lymphocytes. However, there is also another type of defence mechanism, which limits the fitness effect of parasites without preventing or controlling infection. This type of defence is known as infection tolerance (Box 1).

**Box 1. What Is Tolerance, and How Can We Study It?**

Tolerance to parasites, or infection tolerance (not to be confused with immunological tolerance [15]), is the ability of a host to limit the health or fitness effect of a given infection intensity (where infection intensity refers to the number or density of parasites in an individual host). More formally, tolerance is measured as the slope of the relationship between Darwinian fitness (or some proxy of fitness, e.g., a measure of health) and infection intensity. Such a curve, which describes how the value of a phenotypic trait (here health) changes with an environmental factor (here infection intensity), is known as a “reaction norm.” The reaction norm can be estimated within an individual host, or across individuals of a given type. A host type with a shallow slope of the reaction norm has high tolerance, whereas a host type with steep slope has low tolerance. This may seem like an unproblematic definition. Nevertheless, there is often some confusion about what tolerance actually is, and how it relates to other concepts, such as “virulence.” To clarify this, I will here describe one approach to untangle the different factors that may determine the effect of an infection on host health [26].

It seems likely that host health generally decreases in some way with increasing infection intensity; for simplicity we will here assume a linear relationship (Figure 1a). The virulence of an infection (i.e., the reduction in host health or fitness caused by an infection; Figure 1a) depends both on the parasite's ability to grow in and harm the host, and the host's ability to defend itself. Variation in virulence is thus determined by both parasite and host factors. Moreover, both the parasite and the host can, in principle, influence virulence in two different ways: by affecting the infection intensity (i.e., the position on the x-axis in Figure 1a), and by affecting the damage caused by each individual parasite (bacterial cell, virus particle, etc.) (i.e., the slope of the relationship between health and infection intensity in Figure 1a). Thus, if one, for example, is interested in genetic variation for virulence, there are four different sources of variation: host genes affecting infection intensity (resistance), host genes affecting damage per parasite (tolerance), parasite genes affecting infection intensity (often called “exploitation”), and parasite genes affecting damage per parasite (which can be called “per parasite pathogenicity” [26]). It seems likely that several or all of these four factors will contribute to variation in virulence. Their relative importance can be determined by a fully factorial infection experiment, where a number of host genotypes are infected by a number of parasite genotypes [26],[27]. If different host genotypes infected by a given parasite genotype have different infection intensities, this means there is variation in resistance (Figure 1b). If different host genotypes infected by a given parasite genotype have different reaction norms, this means variation in tolerance (Figure 1c). If different parasite genotypes infecting a given host genotype have different infection intensities, then there is variation in “exploitation” (Figure 1d). Finally, if different parasite genotypes infecting a given host genotype have different reaction norms, this shows there is variation in “per parasite pathogenicity” (Figure 1e).

Thus, tolerance is a host trait that explains variation in the relationship between health and infection intensity. It is not just the flip side of virulence; instead it is one of several factors determining the virulence of an infection. Tolerance is best demonstrated by comparing different host types infected by a particular parasite [4]. Variation in tolerance can occur at many levels; among individuals, genotypes, phenotypes, populations, and species.

**Figure 1:**
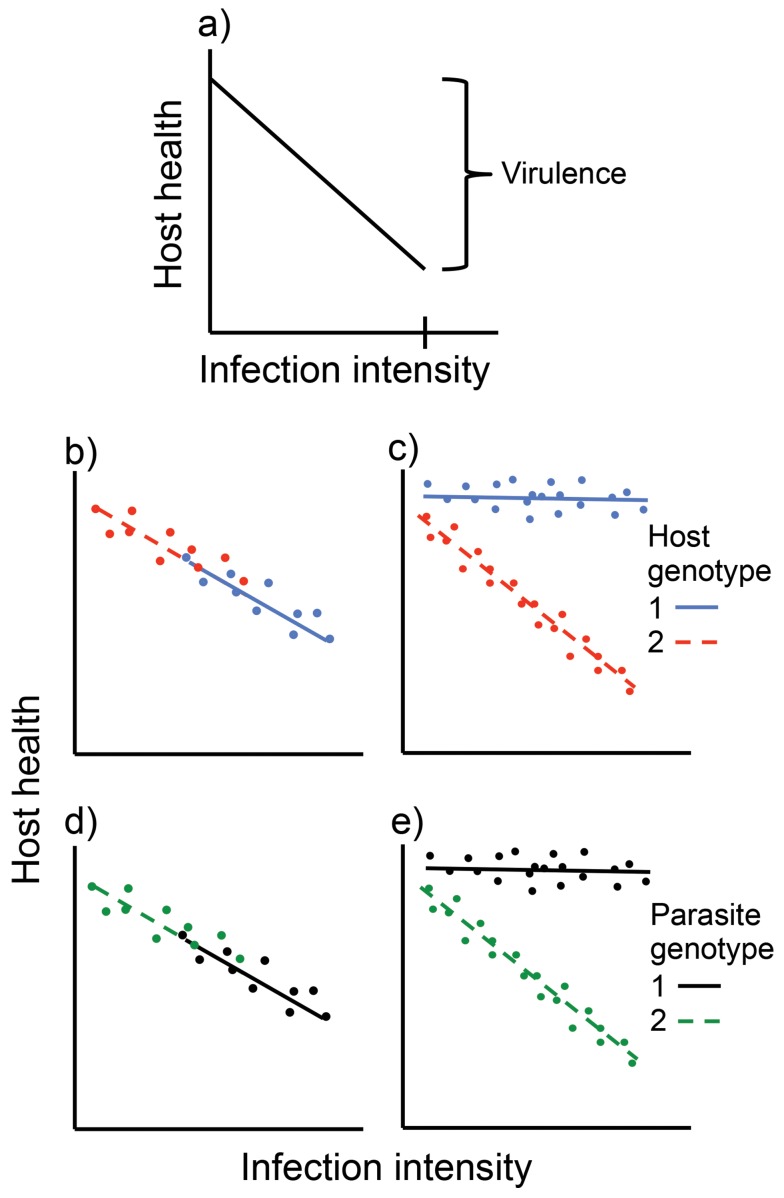
Schematic illustration of how to separate the different host and parasite sources of genetic variation for virulence. Dots are individual hosts. Lines represent reaction norms for different host or parasite genotypes. a) Virulence is the reduction in health a host experiences when infected. It is a function of the infection intensity (the realized infection intensity in this case is indicated with a vertical line on the x-axis) and the slope of the relationship between health and infection intensity. b) Variation among host genotypes for resistance. c) Variation among host genotypes for tolerance. d) Variation among parasite genotypes for “exploitation.” e) Variation among parasite genotypes for “per parasite pathogenicity.”

Both resistance and tolerance are host traits that have evolved to alleviate the health and fitness effects of infection, but they represent two fundamentally different strategies to deal with parasites. The crucial difference is that resistance reduces the risk of infection and/or the replication rate of the parasite in the host, whereas tolerance does not (but note that tolerance can still impose selection on the parasite [3]). It is important to distinguish between resistance and tolerance because these two types of defence lead to different ecological and evolutionary interactions between hosts and their parasites [3]–[6]. For example, if hosts evolve resistance, this should reduce the prevalence of the parasite in the host population. If hosts evolve tolerance instead, this will have a positive effect on parasite prevalence [4].

The study of tolerance to parasites has a long tradition in plant science, where phenomenological studies have documented extensive genetic variation for tolerance at the phenotypic level [7]–[9]. More recently, tolerance has been shown to play a role in several animal host–parasite systems, e.g., [10]–[12]. The case of simian immunodeficiency virus (SIV) in monkeys provides a particularly striking example of the potential significance of tolerance in animals. In macaques—a non-natural host used as an experimental model of HIV infection—the virus replicates readily and infected animals develop AIDS. In contrast, in sooty mangabeys—one of the natural hosts of SIV—the virus also reaches high titres, yet sooty mangabeys remain healthy and do not develop AIDS [13]. Apparently, the sooty mangabey, which has a long history with SIV, has defeated the threat from this virus by evolving tolerance rather than resistance. Taken together, these studies show that tolerance can also be an important component of defence against parasites in animals. However, many important questions remain to be answered to understand the evolution of tolerance.

One of the most pressing questions is, what is the genetic and physiological basis of variation in tolerance? Identifying genes and physiological mechanisms affecting tolerance is a key step to understanding the evolution of this type of defence and could also suggest novel treatments of infectious diseases. Genes involved in tissue repair and scavenging of damaging molecules produced during infection are prime candidates. Factors regulating immune responses, and thereby limiting immunopathology, likely also play an important role [14],[15]. It is important to recognize that a given defence mechanism can potentially affect both resistance and tolerance; as an example, antibodies against a pathogen will affect resistance, but antibodies to a toxin may affect tolerance instead. Infection experiments with knock-out mice and flies have identified some genes influencing tolerance [16],[17]. However, there is still very limited information about what genes and physiological mechanisms contribute to variation in tolerance among individuals, populations, and species.

Another key question is, how does natural selection act on tolerance? Resistance to pathogens is often assumed to not only have fitness benefits but also carry some kind of cost. Such costs have two important consequences: first, they can result in stabilizing selection for an intermediate optimum, and thereby constrain the magnitude of resistance. Second, they can lead to balancing selection that maintains genetic variation for resistance. Should we expect a similar scenario for tolerance, or are tolerance mechanisms generally less costly and therefore under more consistent positive directional selection? If the latter is true, tolerance should evolve more rapidly and have lower levels of genetic variation than resistance.

Three recent studies published in *PLOS Biology* have addressed these questions. Regoes et al. [18] used a candidate gene approach to find genes affecting tolerance to HIV. The primary pathologic effect of an HIV infection is a gradual decrease in the number of CD4+ T cells (T helper cells), which eventually leads to the development of AIDS. The rate of decrease of CD4+ T cells is dependent on the viral load during the chronic phase of the infection. The viral load varies considerably among individuals, and individuals with high viral load generally lose CD4+ T cells faster than individuals with low viral load. It is well known that variation in viral load is partly determined by an MHC class I gene (HLA-B) [19]. Hence, HLA-B influences resistance to HIV. Regoes et al. used data from the Swiss HIV cohort study to investigate if HLA-B also affects tolerance to HIV. They measured tolerance as the relationship between loss of CD4+ T cells and viral load. The key findings were that tolerance varied among HLA-B genotypes, and that HLA-B heterozygotes had higher tolerance than homozygotes. Interestingly, there was no significant effect of particular HLA-B alleles on tolerance. Instead, it appears to be the combination of alleles at this locus that matters. This result contrasts with the effect of HLA-B on resistance, where previous studies have identified specific alleles that limit viral load [19]. Besides genetic effects on tolerance, there was also a significant effect of age, in that tolerance was lower in older individuals.

This is the first study to identify the molecular basis of naturally segregating genetic variation for tolerance. Identifying the genetic basis of not only resistance but also tolerance will improve our understanding of the effects of parasite-mediated selection on host evolution. MHC genes are highly polymorphic and often used as model genes for studying the evolutionary consequences of host–parasite interactions. Such studies have until now been based on the assumption that MHC affects resistance. If MHC also affects tolerance, this means there is an additional selection pressure that needs to be taken into account to explain the evolution of diversity at this gene complex. A key topic for future research is therefore to investigate the relative effect of MHC genes on resistance and tolerance. The results of Regoes et al. also have medical implications; a better understanding of the factors affecting tolerance to HIV could suggest novel treatments. Another key question is therefore to investigate the mechanistic basis of the relationship between HLA-B genotype and tolerance. Regoes et al. hint that HLA-B genotype may affect immunopathology, but the exact mechanism remains to be determined.

Jackson et al. [20] investigated the physiological basis of variation in tolerance to macroparasites (fleas, ticks, and tapeworms) in wild field voles. They measured tolerance as the relationship between body condition and overall parasite load across individuals of different age and sex classes. In adult males, body condition improved with parasite load, whereas in juveniles there was no such relationship. Jackson et al. interpreted this as showing that adult males had higher tolerance than young ones. To identify physiological mechanisms mediating this difference in tolerance between age classes, they also measured expression of a set of immunity genes. Expression of one of these genes—Gata3, a transcription factor involved in Th2 immunity—mirrored the age difference in tolerance; Gata3 expression increased with parasite load in adult males but decreased with load in juveniles. The causal relationship between parasite load and Gata3 expression in adults was confirmed by analyses of longitudinal data. Thus, Gata3 appears to mediate the difference in tolerance between adult and juvenile males. This ambitious study is the first to identify an immunological mechanism influencing tolerance in the wild. The identification of Gata3 fits with the known role of Th2 immunity in tissue repair. However, the reason why infection has different effects on Gata3 expression, and thereby on tolerance, in different age classes is unclear.

An interesting pattern in the field vole study is that there was no overall negative relationship between host condition and parasite load. Thus, based on this measure, the relationship between field voles and these parasites appears to be commensalistic rather than parasitic. However, the maintenance of body condition in face of increasing parasite load was accompanied by decreased investment in reproduction in adult males, indicating that the parasites really had a negative effect on some aspect of host fitness. These results highlight that it is often necessary to consider several different aspects of host health and fitness to get a true picture of the nature of host–parasite interactions.

Hayward et al. [21] measured selection on tolerance to intestinal worms in wild sheep. They used longitudinal data and estimated tolerance as the relationship between body condition and parasite load within an individual. The slope of this relationship varied considerably among individuals, demonstrating phenotypic variation in tolerance. Selection analyses showed that individuals with high tolerance had higher lifetime reproductive success than individuals with low tolerance; thus, tolerance was under positive directional selection. Moreover, pedigree-based analyses indicated that there was little heritable genetic variation for tolerance. Instead, most of the variation in tolerance appears to be environmentally induced.

There have been some previous analyses of selection on tolerance to herbivores in plants, but this is the first study of selection on tolerance to pathogens in animals. The pattern with positive directional selection and lack of genetic variation supports basic models of evolution of tolerance [4],[22], although more recent models suggest that other scenarios are also possible [23]. Further empirical studies of other systems are now desirable to evaluate the generality of Hayward et al.'s result. Future studies should also analyse the shape of selection on tolerance in more detail and test not only for directional but also for stabilizing selection, as has been done for resistance [24]. Moreover, it would be extremely interesting to analyse selection on tolerance and resistance simultaneously. According to theory, tolerance and resistance should be under correlational selection, that is, natural selection should favour specific combinations of resistance and tolerance (high tolerance and low resistance, or low tolerance and high resistance, or intermediate values of both [25]). This is because tolerance and resistance are mutually redundant traits; an individual that is completely resistant cannot gain fitness by increasing its tolerance, and vice versa. Such correlational selection has been demonstrated in plants [25], but remains to be investigated in animals.

These three studies shed light on the genetics, physiology, and ecology of tolerance. They also illustrate the different approaches that can be taken to measure tolerance in natural populations. The critical issue is how to estimate the relationship between fitness or health and parasite load for a given host type (the reaction norm; Box 1). Regoes et al. [18] measured tolerance of a given HLA-B genotype by estimating the relationship between disease progression and viral load across individuals of that genotype. This is analogous to previous studies of plants and animals that have estimated reaction norms for different clones or families. Jackson et al. [20] used the same approach, except that they estimated reaction norms for different age classes instead of genotypes. In contrast, Hayward et al. [21] estimated reaction norms for each individual sheep by using longitudinal data. Longitudinal data is often hard to obtain, but when possible, the individual approach has several advantages. Besides that it facilitates studies of selection (as demonstrated by Hayward et al.), it would also make it easier to investigate the genetic basis of tolerance, for example through genome-wide association studies (GWAS). Clearly, different operational definitions of tolerance are useful in different systems. The crucial thing is that tolerance is defined so that it is not confounded with other host and parasite factors affecting virulence (Box 1). The present studies show that it is possible to achieve this in natural populations, something that opens up for further exciting studies on this topic.

## References

[1] Woolhouse MEJ, Gowtage-sequeria S (2005) Host range and emerging and reemerging Pathogens. Emerg Inf Dis 11: 1842–1847. 10.3201/eid1112.050997 PMC3367654 16485468

[2] WHO (2013) The top 10 causes of death. Available: http://who.int/mediacentre/factsheets/fs310/en/index1.html. Accessed 7 November 2013.

[3] Vale PF, Fenton A, Brown SP (2014) Limiting damage during infection: lessons from infection tolerance for novel therapeutics. PLoS Biol 12: e1001769. 24465177 10.1371/journal.pbio.1001769 PMC3897360

[4] Roy BA, Kirchner JW (2000) Evolutionary dynamics of pathogen resistance and tolerance. Evolution 54: 51–63. 10937183 10.1111/j.0014-3820.2000.tb00007.x

[5] Rausher MD (2001) Co-evolution and plant resistance to natural enemies. Nature 411: 857–864. 11459070 10.1038/35081193

[6] Best A, White A, Boots M (2014) The coevolutionary implications of host tolerance. Evolution; international journal of organic evolution 68: 1426–1435. 24475902 10.1111/evo.12368

[7] Caldwell RM, Schafer JF, Compton LE, Patterson FL (1958) Tolerance to cereal leaf rusts. Science 128: 714–715. 17811449 10.1126/science.128.3326.714

[8] Schafer JF (1971) Tolerance to plant disease. Ann rev phytopathol 9: 235–252.

[9] Cobb NA (1894) Contributions to an economic knowledge of Australian rust (Uredinae) Chap. 10. Agr Gaz N S W 5: 239–250.

[10] Råberg L, Sim D, Read AF (2007) Disentangling genetic variation for resistance and tolerance to infectious diseases in animals. Science 318: 812–814. 17975068 10.1126/science.1148526

[11] Mazé-Guilmo E, Loot G, Páez DJ, Lefèvre T, Blanchet S, et al (2014) Heritable variation in host tolerance and resistance inferred from a wild host – parasite system. Proc R Soc B 281: 20132567. 10.1098/rspb.2013.2567 PMC3924068 24478295

[12] Howick VM, Lazzaro BP (2014) Genotype and diet shape resistance and tolerance across distinct phases of bacterial infection. BMC evol biol 14: 56. 24655914 10.1186/1471-2148-14-56 PMC3997931

[13] Chahroudi A, Bosinger SE, Vanderford TH, Paiardini M, Silvestri G (2012) Natural SIV hosts: showing AIDS the door. Science 335: 1188–1193. 22403383 10.1126/science.1217550 PMC3822437

[14] Råberg L, Graham AL, Read AF (2009) Decomposing health: tolerance and resistance to parasites in animals. Phil trans R Soc Lond B 364: 37–49. 18926971 10.1098/rstb.2008.0184 PMC2666700

[15] Medzhitov R, Schneider DS, Soares MP (2012) Disease tolerance as a defense strategy. Science 335: 936–941. 22363001 10.1126/science.1214935 PMC3564547

[16] Seixas E, Gozzelino R, Ferreira A, Silva G, Larsen R, et al (2009) Heme oxygenase-1 affords protection against noncerebral forms of severe malaria. Proc Natl Acad Sci USA 106: 15837–15842. 19706490 10.1073/pnas.0903419106 PMC2728109

[17] Ayres JS, Schneider DS (2008) A signaling protease required for melanization in Drosophila affects resistance and tolerance of infections. PLoS Biol 6: 2764–2773. 19071960 10.1371/journal.pbio.0060305 PMC2596860

[18] Regoes R, McLaren PJ, Battegay M, Bernasconi E, Calmy A, et al (2014) Disentangling human tolerance and resistance against HIV. PLoS Biol 12: e1001951. 25226169 10.1371/journal.pbio.1001951 PMC4165755

[19] Goulder PJR, Watkins DI (2008) Impact of MHC class I diversity on immune control of immunodeficiency virus replication. Nat rev Immunol 8: 619–630. 18617886 10.1038/nri2357 PMC2963026

[20] Jackson JA, Hall AJ, Friberg IM, Ralli C, Lowe A, et al (2014) An immunological marker of tolerance to infection in wild rodents. PLoS Biol 12: e1001901. 25004450 10.1371/journal.pbio.1001901 PMC4086718

[21] Hayward AD, Nussey DH, Wilson AJ, Berenos C, Pilkington JG, et al (2014) Natural selection on individual variation in tolerance of gastrointestinal nematode infection. PLoS Biol 12: e1001917. 25072883 10.1371/journal.pbio.1001917 PMC4114752

[22] Miller MR, White A, Boots M (2005) The evolution of host resistance: tolerance and control as distinct strategies. J theoret biol 236: 198–207. 16005309 10.1016/j.jtbi.2005.03.005

[23] Best A, White A, Boots M (2008) Maintenance of host variation in tolerance to pathogens and parasites. Proc Natl Acad Sci USA 105: 20786–20791. 19088200 10.1073/pnas.0809558105 PMC2634923

[24] Stjernman M, Råberg L, Nilsson J-Å (2008) Maximum host survival at intermediate parasite infection intensities. PLoS ONE 3: e2463. 18560544 10.1371/journal.pone.0002463 PMC2413421

[25] Fornoni J, Núnez-Farfán J, Valverde PL, Rausher MD (2004) Evolution of mixed strategies of plant defense allocation against natural enemies. Evolution 58: 1685–1695. 15446423 10.1111/j.0014-3820.2004.tb00454.x

[26] Råberg L, Stjernman M (2012) The evolutionary ecology of infectious disease virulence. Ecoimmunology. New York: Oxford University Press. pp. 548–578.

[27] De Roode JC, Altizer S (2010) Host-parasite genetic interactions and virulence-transmission relationships in natural populations of monarch butterflies. Evolution 64: 502–514. 19796153 10.1111/j.1558-5646.2009.00845.x

